# Correction to: Much ado about something: a response to “COVID-19: underpowered randomised trials, or no randomised trials?”

**DOI:** 10.1186/s13063-021-05932-z

**Published:** 2021-12-22

**Authors:** Noah A. Haber, Sarah E. Wieten, Emily R. Smith, David Nunan

**Affiliations:** 1 Meta-Research Innovation Center at Stanford (METRICS), Stanford University, 1265 Welch Rd Palo Alto, Stanford, CA 94305 USA; 2 Department of Global Health, Milken Institute School of Public Health, George Washington University, 950 New Hampshire Avenue, Washington, DC 20052 USA; 3 Centre for Evidence Based Medicine, University of Oxford, Radcliffe Primary Care Building, Radcliffe Observatory Quarter, Woodstock Road, Oxford, OX2 6GG UK

**Correction to: Trials 22, 780 (2021)**

**https://doi.org/10.1186/s13063-021-05755-y**

Following the publication of the original article [1], we were notified of the below correction in attribution of a quote, as well as a missing citation:

Original:

- “For example, Brookes and Butler 2021 inaccurately claim that ‘the Danish mask study showed the overall effects from mask wearing and social distancing were modest.”

Correction:

- “For example, Oraby et al., 2021 inaccurately claim that ‘the Danish mask study showed the overall effects from mask wearing and social distancing were modest.” [citation: 10.1038/s41598-021-82873-2]

The original article has been corrected.
